# Case Report: Autoimmune Hemolysis Anemia After Dihydroartemisinin and Piperaquine for Uncomplicated *Plasmodium falciparum* Malaria

**DOI:** 10.3389/fmed.2021.756050

**Published:** 2022-01-17

**Authors:** Marion Louvois, Loïc Simon, Christelle Pomares, Pierre-Yves Jeandel, Elisa Demonchy, Michel Carles, Pascal Delaunay, Johan Courjon

**Affiliations:** ^1^Rheumatology, Université Côte d'Azur, CHU Nice, Nice, France; ^2^Parasitology and Mycology Department, Université Côte d'Azur, CHU Nice, Nice, France; ^3^Université Côte d'Azur, INSERM 1065, C3M, Nice, France; ^4^Internal Medicine, Université Côte d'Azur, CHU Nice, Nice, France; ^5^Infectious Diseases Department, Université Côte d'Azur, CHU Nice, Nice, France

**Keywords:** hemolytic anemia, malaria, artemisinin-based combination, artemisinin derivatives, autoimmune, direct antiglobulin test

## Abstract

Malaria is still an endemic disease in Africa, with many imported cases in Europe. The standard treatment is intravenous artesunate for severe malaria and oral artemisinin-based combination therapy (ACT) for uncomplicated malaria. Delayed hemolytic anemia (DHA) after intravenous artesunate has been extensively described, and guidelines recommend biological monitoring until 1 month after the end of the treatment. A link with an autoimmune process is still unsure. Nevertheless, cases with positive direct antiglobulin test (DAT) have been reported. Conversely, DHA is not recognized as an adverse effect of oral ACT. Previously, only few cases of DHA occurring after oral ACT without intravenous artesunate administration have been reported. We report the case of a 42-year-old man returning from Togo. He was treated with dihydroartemisinin/piperaquine combination for uncomplicated *Plasmodium falciparum* malaria, with low parasitemia. Nine days after the end of the treatment, the patient developed hemolytic anemia with positive DAT. Eventually, the patient recovered after corticotherapy. After excluding common causes of autoimmune hemolytic anemia, we considered that dihydroartemisinin/piperaquine treatment was involved in this side effect.

## Introduction

Malaria is a mosquito-borne infectious disease endemic in tropical and sub-tropical areas. It is estimated that every year, 12,000–15,000 cases of malaria are imported into European Union countries (1). Among malaria parasites, which can affect humans, *Plasmodium falciparum* is the most common and is responsible for severe malaria and death. The recommended treatment for severe malaria is intravenous artesunate, whereas, for uncomplicated *P. falciparum* malaria, the treatment is oral artemisinin-based combination therapy (ACT) (2).

The risk of delayed hemolytic anemia (DHA) after intravenous artesunate is well-acknowledged (3). DHA frequently occurs between days 7 and 21 after the initiation of treatment. However, the pathophysiology of this adverse event has not been entirely deciphered. Artesunate-induced splenic pitting of parasitized erythrocyte is the main cause: (3): some dead trophozoites are turned into pyknotic forms within the erythrocytes (4), these latest being damaged have a shorter life span (7-21 days) and are cleared by the spleen. More recently, another mechanism of anemia has been described, corresponding to drug-induced immune hemolytic anemia (4). This is supported by the detection of positive direct antiglobulin test (DAT) after artesunate treatment during DHA management, and by the effectiveness of corticotherapy (4–6). Guidelines recommend hemoglobin monitoring until 1 month after the end of the treatment, but to date, DAT is not required.

Conversely, DHA is not commonly recognized as an adverse effect of oral ACT. To our knowledge, only five cases that occurred after oral ACT have been reported (7); one of which is associated with positive DAT (8). Recently, Kurth et al. have described in a prospective study that hemolysis after oral ACT is more frequent than previously suspected (9).

Herein, we present one case of plausible autoimmune hemolyticanemia after dihydroartemisinin and piperaquine treatment for uncomplicated *P. falciparum* malaria.

## Case Presentation

On December 31, 2019, a 42-year-old Caucasian male patient visited the emergency department of Nice University Hospital for abdominal pain. His medical history was marked by cured colon cancer 10 years ago, and uncomplicated *P. falciparum* malaria with 4% parasitemia, diagnosed on December 13, 2019, 10 days after returning from Togo. He was treated with 3 days of dihydroartemisinin/piperaquine. On December 16 and 24, hemoglobin level was 10.8 and 10.5 g/dl, respectively. The treatment was completed 15 days before his visit to the emergency department. It is important to notice that parasitemia was below 0.025% on day 3 of treatment and was negative on day 11. At the emergency department, clinical examination showed left hypochondrial pain and cutaneous mucosal pallor. The blood test showed macrocytic anemia at 5.4 g/dl, with hemolysis criteria. Indeed, bilirubin was high (45 μmol/l), haptoglobin was under 0.1 g/l, LDH level was 2,835 U/L and reticulocytes were at 297 g/L. Parasitemia was negative. The CT scan revealed splenomegaly with some areas of splenic infarction. The patient was then hospitalized in the infectious disease unit in order to investigate the origin of this hemolytic anemia. Malaria parasitemia control was negative. DAT was positive for anti-C3d, leading to the diagnosis of cold autoimmune hemolytic anemia.

The patient was a Caucasian male without a family history of anemia or hemolysis such as Sickle cell disorders, his diet did not include food/herbal medicine neither in Togo nor in France. A workup for autoimmune hemolytic anemia causes was made. Autoimmune assays (antinuclear antibodies, ANAs, extractable nuclear antigen, ENA, rheumatoid factor) were negative. Blood protein electrophoresis and immunofixation were normal. The lymphocyte immunophenotyping performed in November 2021 (2 years after) was normal. EBV, hepatitis B, C, and E, HIV, HTLV1, *Chlamydia pneumonia*, and *Coxiella burnetti* serological tests were negative. CMVIgM and IgG antibodies were both positive, but viral DNA detection in the blood by PCR was negative. *Mycoplasma pneumoniae* serology showed that IgM and IgG antibodies were positive. Nevertheless, the serological monitoring did not show seroconversion and therefore did not support an acute infection. Finally, Parvovirus B19 IgM and IgG antibodies were close to the positive cutoff, but the patient did not have any consistent symptoms (no flu-like symptoms and no lymphocytosis). *Mycoplasma pneumonia*, Parvovirus B19, and CMV serologies were considered non-specific reactions. Hemoglobin electrophoresis analysis and G6PD testing were within the normal range.

We finally concluded that an autoimmune process could be involved in this hemolytic anemia following dihydroartemisinin/piperaquine treatment complicated by splenic infarction. At first, red blood cell transfusion was performed before the positive DAT was available and failed to improve the level of hemoglobin, which stayed close to 6 g/dl. Then, an intravenous corticosteroid therapy (methylprednisolone) at 1 mg/kg was started, and hemoglobin increased to 8.3 g/dl in 3 days, and hemolysis markers resolved. The patient was discharged with oral corticosteroid therapy for 3 weeks. On follow-up red blood cell count 2 weeks after he was discharged, hemoglobin was 11.4 g/dl, haptoglobin was 0.22, LDH was 786 U/L, and bilirubin was 9 μmol/L. A follow-up CT scan 1 month later showed a decrease in splenomegaly with the persistence of a splenic infarct area.

## Discussion

Autoimmune hemolytic anemia (AIHA) is acquired hemolysis due to the immune system of the host acting against its own red cell antigens. Hemolysis is suggested by normo/macrocytic anemia, the elevation of reticulocytes LDH, and unconjugated bilirubin, and low haptoglobin value. Then, AIHA is based on positive DAT without any alternative origin. When DAT is positive for IgG or IgG + C3d, a diagnosis of warm AIHA can be made. Cold agglutinin syndrome would reveal positive anti-C3d only. Among AHAI, approximately 65% of patients have warm AHAI, which is mainly caused by autoimmune disease and hematologic malignancy, while cold AHAI is mainly caused by infectious disease and hematologic malignancy (10). Drug-dependent hemolytic anemia is another potential cause of AIHA.

Hemolytic anemia is a common finding in acute malaria, especially severe malaria (11). It may persist for weeks after the elimination of parasites (12). The underlying causes are multifactorial: the destruction of infected red blood cells, clearance of uninfected red blood cells, erythropoietic suppression, and dyserythropoiesis (13). Nevertheless, our patient presented uncomplicated malaria with low parasitemia and hemoglobin level of 10.8 g/dl at the end of dihydroartemisinin/piperaquine treatment but still developed severe hemolytic anemia 8 days after complete elimination of parasites.

The risk of delayed hemolytic anemia (DHA) after intravenous artesunate treatment is well-acknowledged; more recently, artesunate-induced immune hemolytic anemia and a putative mechanism have also been reported (4). Most of the drug-induced autoimmune hemolytic anemia is warm AHAI, which is not the case for our patients (14). Nevertheless, a retrospective study on post artesunate-delayed hemolysis described four cases of AHAI with positive DAT for C3d only (4), and three other similar cases can be found in the literature (6, 15, 16). Conversely, DHA secondary to oral ACT has been less reported. In the Kurth et al. prospective study in which 20 patients were included, eight treated with oral ACT presented posttreatment hemolysis, but it mainly occurred at the subclinical level and no patient with anemia had a positive DAT (9). A very recent systematic review (7) described 5 cases of DHA secondary to treatment with oral ACT only, including the only previously published with positive DAT (8).

Two mechanisms of DHA after treatment by artesunate are described: the pitting process, which is the most frequent; and more rarely, drug-induced autoimmune hemolytic anemia. This second etiology has been suggested by the increasing number of cases with positive DAT and corticosteroids effectiveness (4).

Among the cases of DHA after oral ACT, the one with a positive DAT recovered with oral prednisone for 1 month, and the other recovered without corticotherapy (7). Positive DAT may be considered by some authors as a non-specific marker of the immune response following malaria (7). However, careful description of those cases, including DAT results, during the following years is mandatory, because there are some therapeutic issues that need to be solved with the use, or non-use, of corticosteroids.

According to the French guidelines, atovaquone-proguanil is the second-line treatment for uncomplicated *P. falciparum* malaria, and quinine is the third-line treatment (17). Therefore, in our case, atovaquone-proguanil should be proposed to the patient in case of a new uncomplicated *P. falciparum* malaria episode.

Regarding the splenic infarction, it can lead to this complication (18). In our case, hemoglobinopathies, viral infections, emboligenic disorders, and malignancy were not involved. However, the biological tests for a coagulation disorder such as antiphospholipid syndrome or thrombophilia has not been performed. The physiopathology of splenic infarction in malaria remains poorly understood. The literature describes several hypotheses: hypercoagulopathy, high level of parasitemia and microvascular sequestration of parasitizederythrocytes, and acute splenic enlargement (18). Anemia is one of the main risk factors for splenic infarction. Therefore, our patient's splenic infarction could be secondary to malaria itself, worsened by hemolytic anemia. Indeed, red blood cell destruction can cause an increased release of vasoactive cytokines or microthrombus (19).

## Conclusion

To our knowledge, our case report is the first to describe DHA in a patient treated exclusively with dihydroartemisinin/piperaquine and linked to an autoimmune process. A diagnosis of DHA after dihydroartemisinin/piperaquine was made after excluding the differential diagnosis. Taken together, the positive DAT, the futility of red blood cell transfusion, and the efficacy of corticosteroid therapy in our patient are strong arguments to link DHA to an autoimmune process. Hemolysis monitoring after dihydroartemisinin/piperaquine should be reassessed, even in cases of uncomplicated malaria with low parasitemia. In cases of DHA, clinicians should look for an autoimmune process in order to discuss corticosteroid prescription.

## Data Availability Statement

The original contributions presented in the study are included in the article/Supplementary Material, further inquiries can be directed to the corresponding author/s.

## Author Contributions

JC, ED, and P-YJ made the diagnosis and interpreted the data. JC and ML conceived the research project, wrote the manuscript, and performed a literature review. CP, PD, LS, and MC helped design the manuscript and synthesize the biological test protocols. All the authors read and approved the final version of the manuscript.

## Conflict of Interest

The authors declare that the research was conducted in the absence of any commercial or financial relationships that could be construed as a potential conflict of interest.

## Publisher's Note

All claims expressed in this article are solely those of the authors and do not necessarily represent those of their affiliated organizations, or those of the publisher, the editors and the reviewers. Any product that may be evaluated in this article, or claim that may be made by its manufacturer, is not guaranteed or endorsed by the publisher.
